# Genotyping and drug susceptibility profiling of *Prototheca* sp. strains isolated from cases of protothecosis in dogs

**DOI:** 10.1111/jvim.17173

**Published:** 2024-11-20

**Authors:** Angelika Proskurnicka, Mateusz Iskra, Sylwia Wronka, Zofia Bakuła, Patrizia Danesi, Marconi Rodrigues de Farias, Fábio Vinícius Ramos Portilho, Márcio Garcia Ribeiro, Uwe Rösler, Rui Kano, Richard Malik, Tomasz Jagielski

**Affiliations:** ^1^ Department of Medical Microbiology, Institute of Microbiology, Faculty of Biology University of Warsaw Warsaw Poland; ^2^ Istituto Zooprofilattico Sperimentale delle Venezie Padua Italy; ^3^ Postgraduate Program in Animal Science Pontifical Catholic University of Paraná Curitiba Brazil; ^4^ Department of Animal Production and Preventive Veterinary Medicine São Paulo State University Botucatu Brazil; ^5^ Institute for Animal Hygiene and Environmental Health Freie Universitaet Berlin Berlin Germany; ^6^ Department of Veterinary Dermatology Nihon University School of Veterinary Medicine Fujisawa Kanagawa Japan; ^7^ Centre for Veterinary Education, Sydney School of Veterinary Science The University of Sydney Sydney Australia

**Keywords:** algae, colitis, *cytb*, dog, *Prototheca* spp., systemic infection

## Abstract

**Background:**

Protothecosis in dogs is a rare, yet emerging disease, distinguished by its often‐aggressive clinical course and high fatality rate. Our study was conducted to enhance treatment protocols for affected dogs by better understanding the genetic diversity and drug resistance patterns of *Prototheca* species.

**Objectives:**

To identify species and drug susceptibility profiles of an international collection of 28 *Prototheca* strains isolated from cases of protothecosis in dogs.

**Animals:**

None.

**Methods:**

Retrospective study. Species‐level identification was made for isolates from 28 dogs in 6 countries by molecular typing with the partial *cytb* gene as a marker. For the determination of minimum inhibitory concentrations (MICs) and minimum algicidal concentrations (MACs), the Clinical Laboratory Standards Institute (CLSI) protocol (M27‐A3) was used.

**Results:**

*Prototheca bovis* was the most prevalent species, accounting for 75% (21/28) of the cases, followed by *P. wickerhamii* (18%; 5/28) and *P. ciferrii* (7%; 2/28). Of the 6 drugs tested, efinaconazole (EFZ) was the most potent *in vitro*, with its median MIC and MAC values equal to 0.125 mg/L. The lowest activity was found for fluconazole (FLU), with MIC and MAC medians of 48 mg/L and 64 mg/L, respectively.

**Conclusions and Clinical Importance:**

Our study identifies *P. bovis* as the species that most frequently causes protothecosis in dogs, which suggests the possibility of cross‐species infection from other animals, especially cows. Additionally, it indicates that EFZ could be used in the treatment of infection in the colon.

**Abbreviations**
AMB: amphotericin B
CLSI: Clinical Laboratory Standards Institute
DMSO: dimethyl sulfoxide
EFZ: efinaconazole
FDA: Food and Drug Administration
FLU: fluconazole
ITZ: itraconazole
KTZ: ketoconazole
MACs: Minimum Algicidal Concentrations
MICs: Minimum Inhibitory Concentrations
RVZ: ravuconazole

## 1 INTRODUCTION

*Prototheca* species are unicellular, achlorophyllous, yeast‐like microalgae inhabiting diverse natural environments, including plants, soil, and bodies of water.^1^ Although normally saprophytic, these organisms can act as opportunistic pathogens of both humans and animals, resulting in a variety of diseases, collectively referred to as protothecosis.

*Prototheca* spp. were originally classified as fungi because of their morphological features and lack of chlorophyll.^2^ Since then, several substantial revisions to *Prototheca* taxonomy have been made, with the increasing availability of phenotypic, chemotaxonomic, and molecular data.^3^ The current classification system is based on the mitochondrial *cytb* gene, under which, a total of 18 species are delineated.^3^, ^4^, ^5^ Of those, 4 are reported as the causative agents of protothecosis in dogs, namely *P. bovis*, *P. ciferrii*, *P. wickerhamii*, and *P. zopfii*.^6^, ^7^, ^8^, ^9^, ^10^ The latter, however, has been separated into 2 genotypes, that is, *P. zopfii* gen. 1 and *P. zopfii* gen. 2, which are renamed as *P. ciferrii* and *P. bovis*, respectively.^3^

The 1st case of *Prototheca* infection in a dog was documented in 1969.^11^ Since then, a total of 125 cases of protothecosis in dogs have been reported in 80 published cases up until 2023. The disease is typically characterized by an apparently sudden onset, involvement of multiple organs, and a fatal progression.^6^, ^10^ The problem is further compounded by the lack of standardized therapeutic guidelines for diagnosis or treatment. In general, treatment is largely empiric, with poor predictability and often unsuccessful outcomes. Only a few documented treatment attempts resulted in a full recovery of the animal.^8^, ^12^, ^13^, ^14^ This relates to a hallmark feature of *Prototheca* algae, which is their robust refractoriness to a range of physical and chemical stress conditions. As shown by studies on strains of human, bovine, and environmental origin, *Prototheca* spp. are resistant to a wide spectrum of antimicrobial agents, and there is often no clear correlation between the drug susceptibility testing results assayed *in vitro* and the clinical efficacy of the drug.^15^, ^16^, ^17^

The objective of this work was to perform a combined microbiological analysis of *Prototheca* strains isolated from cases of protothecosis in dogs through identification of the species at a taxonomic level and assessment of antimicrobial susceptibility and nonresistance. Thus, *cytb* gene‐based genotyping and drug susceptibility testing with a panel of 6 antifungal agents, either in the developmental phase or already available on the pharmaceutical market, was performed for an international collection of 28 *Prototheca* sp. strains isolated from as many dogs suffering from protothecosis. Overall, our study provides a large microbiological analysis of protothecosis detected in dogs.

## 2 MATERIALS AND METHODS

### 2.1 Algal strains

A total of 28 *Prototheca* sp. strains were used in our study. They were all originally isolated from dogs from 6 different countries: Germany (n = 8), Brazil (n = 7), Italy (n = 7), Australia (n = 3), Japan (n = 2), and the United Kingdom (n = 1). In addition to isolates from dogs, 4 reference strains, namely *Prototheca bovis* SAG 2021 (T), *Prototheca ciferrii* SAG 2063 (T), *Prototheca wickerhamii* ATCC 16529 (T), and *Pichia kudriavzevii* ATCC 6258 (T), purchased from international culture collections, were used as quality controls for drug susceptibility testing (Table 1).

**TABLE 1:** List of *Prototheca* sp. strains used in the study.

No.	Isolate ID	Identification			Country of origin	Reference
		Original	*cytb*‐based genotyping			
			Outcome	GenBank accession no.		
1.	BP1	*P. zopfii*	*P. bovis*	OQ869619	Brazil	…
2.	BP2	*P. zopfii*	*P bovis*	OQ869620	Brazil	…
3.	Bras14	*P. zopfii* gen. 2	*P. bovis*	OQ869621	Brazil	^14^
4.	Bras23	*P. zopfii* gen. 2	*P. bovis*	OQ869622	Brazil	^18^
5.	DAW	*P. zopfii* gen. 2	*P. bovis*	OQ869636	United Kingdom	…
6.	PBC	*P. bovis*	*P. bovis*	OQ883868	Japan	…
7.	PRO‐EM‐SPC 21M	*P. zopfii*	*P. bovis*	OQ869630	Italy	…
8.	P232	*P. zopfii* gen. 2	*P. bovis*	MF163470	Germany	…
9.	P233	*P. zopfii* gen. 2	*P. bovis*	OQ869623	Germany	…
10.	P280	*P. zopfii* gen. 2	*P. bovis*	OQ869624	Germany	…
11.	P310	*P. zopfii* gen. 2	*P. bovis*	OQ869625	Germany	…
12.	P511	*P. zopfii* gen. 2	*P. bovis*	OQ869626	Germany	…
13.	P528	*P. zopfii* gen. 2	*P. bovis*	OQ869627	Germany	…
14.	P541	*P. zopfii* gen. 2	*P. bovis*	OQ869628	Germany	…
15.	WP1	*P. zopfii*	*P. bovis*	OQ869631	Italy	…
16.	WP2	*P. zopfii*	*P. bovis*	OQ869632	Italy	…
17.	256/2021 UFPR	*P. bovis*	*P. bovis*	OQ869617	Brazil	…
18.	3826	*P. bovis*	*P. bovis*	OQ869629	Italy	^6^
19.	3848	*P. bovis*	*P. bovis*	OQ869615	Australia	^6^
20.	3849	*P. bovis*	*P. bovis*	OQ869616	Australia	^6^
21.	77/ACT‐16	*Prototheca* sp.	*P. bovis*	OQ869618	Brazil	…
22.	WP3	*P zopfii*	*P. ciferrii*	OQ869633	Italy	…
23.	WP4	*P. ciferrii*	*P. ciferrii*	OQ869634	Italy	^6^
24.	Japan 6	*P. wickerhamii*	*P. wickerhamii*	OQ883865	Japan	^19^
25.	P543	*P. wickerhamii*	*P. wickerhamii*	OQ883866	Germany	…
26.	WP5	*P. wickerhamii*	*P. wickerhamii*	OQ869635	Italy	^6^
27.	059/19	*P. wickerhamii*	*P. wickerhamii*	OQ106961	Brazil	^8^
28.	3847	*P. wickerhamii*	*P. wickerhamii*	OQ883867	Australia	^6^
29.	SAG 2021	*P. zopfii* gen. 2	*P. bovis*	MF163469	Germany	^5^
30.	SAG 2063	*P. zopfii* gen. 1	*P. ciferrii*	MF163464	Germany	^5^
31.	ATCC 16529	*P. wickerhamii*	*P. wickerhamii*	MF163459	United States	^5^
32.	ATCC 6258	*Pichia kudriavzevii*	…	…	Sri Lanka	…

The strains were cryopreserved with Viabank Bacterial Storage Beads (MWE Medical Wire, UK) at −70°C and revived by streaking a loopful (10 μL) of the frozen culture onto Sabouraud's Dextrose Agar (SDA; Biomaxima, Poland), and incubated at 30°C aerobically for 72 hours. Subcultures were maintained in the same medium and under the same conditions, as described above.

### 2.2 Species identification

Species‐level identification was made by molecular typing with the partial *cytb* gene as a marker.^5^ Briefly, genomic DNA was obtained with the GeneMATRIX Environmental DNA & RNA Purification Kit (EURx, Poland). For polymerase chain reaction (PCR) amplification, a primer pair cytb‐F1 (5′‐GyGTwGAACAyATTATGAGAG‐3′), and cytb‐R2 (5′‐wACCCATAArAArTACCATTCWGG‐3′), and ColorTaq PCR Master Mix (EURx, Poland) were used, as per manufacturer's instructions. Thermocycling conditions were 3 minutes at 95°C, followed by 35 cycles of 30 seconds at 95°C, 30 seconds at 50°C, and 30 seconds at 72°C, with a final extension of 5 minutes at 72°C. The PCR products were then subjected to PCR‐Restriction Fragment Length Polymorphism (RFLP) analysis, that is, doubly digested with FastDigest RsaI and TaiI enzymes (Thermo Fisher Scientific, USA), under conditions recommended by the supplier, fractionated on 4% agarose gels, and visualized by ethidium bromide (5 mg/L) staining, and exposure to ultraviolet light (UV). The restriction patterns were analyzed, as described elsewhere.^5^

For all strains identified with PCR‐RFLP as *P. bovis*, the PCR products were also purified with Short DNA Clean‐Up (EURx, Poland) and sequenced with the same primers as used for the amplification. This was done to avoid misidentifications of *P. bovis* and *P. ciferrii*.^3^ The assembled sequences were analyzed with the *Prototheca*‐ID web application and deposited in the sequence repository of this application^20^ and the National Center for Biotechnology Information (NCBI) GenBank database (Table 1).

### 2.3 Drug susceptibility testing

In the absence of universally accepted guidelines, specifically applicable to *Prototheca* spp., determination of Minimum Inhibitory Concentrations (MICs) and Minimum Algicidal Concentrations (MACs) was performed by broth microdilution method, in 96‐well microtiter plates (Genos, Poland), pursuant to the Clinical Laboratory Standards Institute (CLSI) protocol (M27‐A3) for drug susceptibility testing of yeast‐like fungi.^21^ The only modification to the protocol was that a suspension of the algal inoculum was adjusted to a 6 McFarland turbidity standard. This adjustment was made in order to obtain a CLSI‐recommended stock suspension concentration, which translates to ca. 1.0 to 5.0 × 10^6^ cfu/mL.

A total of 6 drugs were tested, including amphotericin B (AMB), efinaconazole (EFZ), fluconazole (FLU), itraconazole (ITZ), ketoconazole (KTZ), and ravuconazole (RVZ), all supplied by Sigma‐Aldrich, Poland. Working solutions were prepared in dimethyl sulfoxide (DMSO; BioShop; Canada) immediately before use.

For each *Prototheca* sp. strain, all drugs were tested at doubling concentrations, ranging from 0.031 to 64 mg/L (AMB), 0.002 to 1 mg/L (EFZ), 1 to 128 mg/L (FLU), 1 to 128 mg/L (ITZ), 0.25 to 32 mg/L (KTZ), and 0.004 to 2 mg/L (RVZ), in triplicates. The MIC was described as the lowest concentration of the drug that completely inhibited growth of the *Prototheca* strain, as detected by the naked eye.

The MAC values were determined as reported earlier.^22^ Briefly, after MIC determination, 100‐μL samples taken from wells described as 2‐fold and 4‐fold MICs were spread across the surface of the SDA plates. After 72 hours of incubation at 30°C, the number of colonies was counted. The MAC was defined as the lowest drug concertation that killed at least 99.9% of the algal cells when compared with the control.

Only if 2 replications showed the same result, the isolate was given the final MIC and MAC values.

## 3 RESULTS

All *Prototheca* strains (28) used in our study were isolated from cases of protothecosis in dogs, of which 10 had previously been published between 2006 and 2023 (nos. 3, 4, 18‐20, 23, 24, 26‐28; Table 1 and Table S1). The strains originated from dogs, mostly with systemic disease, living in 6 countries on 4 continents. Selected epidemiologic, laboratory, and clinical details on *Prototheca* sp. isolates under the study are provided in Table S1.

### 3.1 Genotyping

Of 28 strains evaluated, 10 (36%) had previously been identified as: *P. bovis* or *P. zopfii* gen. 2 (5/10; 50%), *P. wickerhamii* (4/10; 40%), and *P. ciferrii* (1/10; 10%). Genotyping performed in our study corroborated the original identification. For the remaining isolates, representing unpublished cases, the species identity was fully corroborated except for 5 *P. zopfii* isolates, of which 4 were identified as *P. bovis*, and 1 as *P. ciferrii*. In addition, 1 *Prototheca* sp. isolate was identified as *P. bovis*.

Overall, 21/28 (75%) isolates were classified as *P. bovis*, 5/28 (18%) as *P. wickerhamii*, and 2/28 (7%) as *P. ciferrii*. The results of the original and confirmatory species identification of *Prototheca* algae under our study are presented in Table 1 and Table S1. In all countries, from which at least 3 strains were available (Australia, Brazil, Germany, and Italy), *P. bovis* was always the most common species, followed by *P. wickerhamii* (Table 1 and Table S1).

### 3.2 Drug susceptibility testing

**FIGURE 1:**
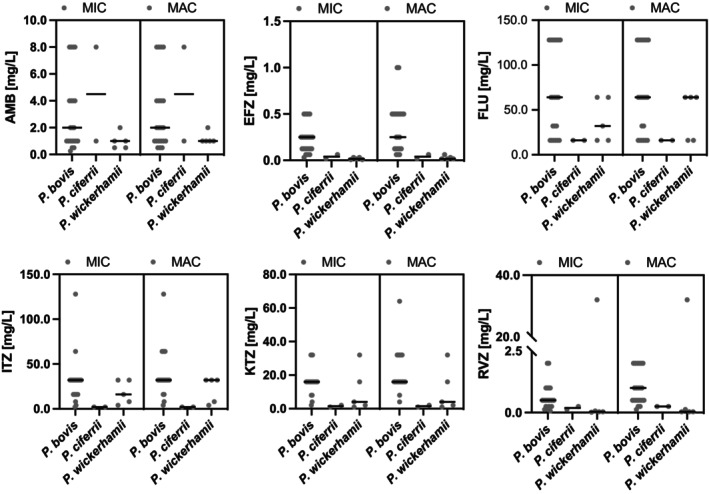
Minimum Inhibitory Concentrations (MICs) and Minimum Algicidal Concentrations (MACs) of drugs tested on 28 *Prototheca* sp. strains. AMB, amphotericin B; EFZ, efinaconazole; FLU, fluconazole; ITZ, itraconazole; KTZ, ketoconazole; and RVZ, ravuconazole.

Of 6 drugs tested, all showed activity against *Prototheca* sp. strains at the concentrations used in our study. The highest MIC and MAC median values were reported for FLU viz 48 mg/L and 64 mg/L, respectively. Likewise, a weak activity against *Prototheca* spp. was demonstrated for ITZ with median MIC and MAC both 32 mg/L. The 3rd least potent antiprotothecal drug was KTZ with its median MIC/MAC values being 16 mg/L. The highest anti‐*Prototheca* activity was shown for EFZ, a novel compound of the triazole series (median MIC/MAC, 0.125 mg/L; range, 0.008‐0.5 mg/L for MICs and 0.016‐0.1 mg/L for MACs). The other 2 drugs that displayed activity toward *Prototheca* algae were RVZ and AMB, with their median MICs of 0.5 mg/L and 1 mg/L, respectively. The median MIC and MAC values were equal for EFZ (0.125 mg/L), ITZ (32 mg/L), RVZ (.5 mg/L), and KTZ (16 mg/L; Table S2 and Figure 1). The algicidal effect was also demonstrated for AMB as its MACs were only slightly higher than MICs (1 and 1.5 mg/L).

## 4 DISCUSSION

Three fourths (21/28) of the *Prototheca* sp. isolates were identified as *P. bovis*. Included in that number were 15 strains originally described as *P. zopfii* (or *P. zopfii* gen. 2). This clearly shows that *P. bovis* is the major etiological agent of protothecosis in dogs. It also implies that wherever in the older literature, *P. zopfii* was stated to be the causative agent (at least 25 published case reports), most likely it would now be reclassified as *P. bovis*.^3^

As in dogs, *P. bovis* is the main etiological agent of protothecal mastitis in cows, which is the most common form of protothecal disease in animals.^23^, ^24^, ^25^ It is thus not surprising that *P. bovis* was also the most frequently isolated *Prototheca* species from the dairy farm environment.^23^ In contrast, *P. wickerhamii* has been the major protothecal pathogen for cats^26^, ^27^, ^28^, ^29^, ^30^ and goats,^31^, ^32^ and humans.^33^, ^34^ It thus seems that there might exist a certain host specificity among *Prototheca* species, and that this specificity might be related, at least to some extent, to the genomic landscape of the pathogen and possibly the host too. This uneven species distribution is observed in the natural environment. Whereas, *P. bovis* is the most frequently isolated *Prototheca* species from the dairy farm surroundings,^23^, ^35^ in aquatic reservoirs *P. wickerhamii*, *P. pringhsheimii*, and *P. cerasi* are the more prevalent species.^4^

The identification method used in our study relies on the mitochondrially‐encoded *cytb* gene, which has the highest discriminatory capacity compared with previously used ribosomal DNA markers, such as the small ribosome subunits (SSU), large ribosome subunits (LSU), and internal transcribed spacers (ITS).^5^ No other typing method except for PCR‐RFLP of the *cytb* gene has been tested and optimized for the differential identification of all *Prototheca* species recognized so far. The *cytb* gene‐based PCR‐RFLP was developed based on the sequencing results for the *cytb* gene, upon which the current *Prototheca* taxonomy was established upon.^4^, ^5^ The ease of use and short turnaround time make the *cytb* gene analysis a gold standard for *Prototheca* speciation.

The environmental ubiquity and persistence of *Prototheca* spp., increasing the risk of being transmitted to animals, is largely attributed to the ability of the algae to survive harsh conditions, including high temperatures or chemical treatments, such as chlorination, which are commonly used in water treatment.^36^, ^37^ *Prototheca* algae exhibit resistance to commonly employed antimicrobial agents, which translates into a lack of efficacy of common medical treatments. Variation in drug efficacy exists across a variety of species is also confirmed by the present study.^16^, ^17^, ^22^, ^34^, ^38^, ^39^, ^40^ Similar to what has recently been demonstrated for human *Prototheca* isolates,^38^ the drug exhibiting the lowest MIC and MAC values against canine isolates was EFZ, followed by RVZ, and AMB. Other azoles shared a similar range of mean MIC and MAC values (ie, 16‐48 mg/L and 16‐64 mg/L, respectively), with the highest values observed for FLU, which is again in line with the findings for *Prototheca* sp. strains from human patients.^38^ Likewise, a general susceptibility hierarchy of the *Prototheca* species was the same as when human and bovine isolates were tested, with *P. bovis* being more resistant than *P. ciferrii*, which in turn was more resistant than *P. wickerhamii*.^15^, ^38^

All drugs that are addressed in our study are approved by the United States Food and Drug Administration (FDA) for clinical use, except for RVZ, although EFZ is only approved for topical treatment of fungal infections of the nails.^41^ RVZ is a novel human triazole drug available in Japan since 2018. It is a broad‐spectrum antifungal agent that exhibits excellent activity against *Candida albicans* and *Cryptococcus neoformans*. The drug is developed as an oral formulation for treating onychomycosis. A prodrug of RVZ called fosravuconazole L‐lysine ethanolate (BFE1224 or F‐RVCZ) has advanced to clinical use. To treat human dermatophyte infections, F‐RVCZ (equivalent to 100 mg RVZ) is given PO once daily for 12 weeks; it showed significantly higher complete cure rates (59.4%) compared with placebo (5.8%) at 48 weeks. The study also found F‐RVCZ to be well‐tolerated, with mostly mild to moderate adverse events.^42^ An earlier phase I/II trial in adults with onychomycosis treated with RVZ 200 mg/day, 100 mg/week and 400 mg/week for 12 weeks revealed steady‐state serum drug concentrations around 3000 ng/mL.^43^ No studies of this drug in the dog have been conducted to the best of our knowledge, however 1 of the authors used this drug (unsuccessfully) to treat a case of disseminated protothecosis in an Australian shepherd domiciled in Florida (personal communication; January, 2024). The drug was well‐tolerated, even though it did not appear to be effective clinically at the dosage used. Blood concentration of the drug was not determined during treatment. Nevertheless, this drug might be useful if given at higher doses as determined by therapeutic drug monitoring aiming to achieve serum concentrations 4 to 5 times the MIC for the individual *Prototheca* strain isolated from the dog. The drug might also be useful if given as a retention enema for treating protothecal colitis.

EFZ is an antifungal medication of the triazole class, used to treat onychomycosis. It is a topical solution applied directly to affected toenails once daily for 48 weeks. It has a broad‐spectrum antifungal activity against dermatophytes (eg, *Trichophyton* species), yeasts (eg, *Candida albicans*), and nondermatophyte molds.^39^, ^44^ To the best of authors' knowledge, EFZ has never been used for the treatment of protothecosis in dogs; the drug is marketed as a topical preparation for treating dermatophyte infections of the nails, and there is no information readily available concerning its use as oral (systemic) treatment, or as topical treatment in the alimentary tract, for example, as a retention enema. It was approved in 2014 for the treatment of onychomycosis. Efinaconazole 10% solution is effective in treating mild to moderate toenail fungal infections, with cure rates around 15%‐18% and mycological cure around 55% after 52 weeks of treatment. Common adverse effects include skin redness, itching, burning, stinging, blisters, and ingrown toenail around the treated nail.^41^, ^45^ We have not been able to find any information about the systemic use of this agent. In addition, the use of low doses of EFZ in combination with other drugs used to treat systemic infections may have synergistic or additive effects against different *Prototheca* species. Therefore, although EFZ seems to be a promising agent, it is not yet suitable for use in cases of multiorgan manifestations of the disease, although it might be suitable for use as a retention enema.

Furthermore, despite exhibiting promising in vitro results, commonly prescribed medications, including those tested in our study, are frequently ineffective in treating protothecal infections in dogs.^10^, ^46^, ^47^, ^48^, ^49^, ^50^, ^51^, ^52^ New potential agents, including nanoparticles, iodinated carbamates, guanidine, or 3‐bromopyruvate, have been evaluated.^22^, ^53^, ^54^ Modifying existing medications, such as cochleated amphotericin B (CAMB) formulations could also be effective.^55^, ^56^

To conclude, *Prototheca* algae and protothecosis have rarely been studied in the context of veterinary medicine. Our study brings to attention this unusual yet emerging disease. It emphasizes the important role of * P. bovis* in the etiology of protothecosis in dogs. It also highlights EFZ and RVZ, novel compounds of the triazole series, as potential treatments for the disease, suggesting their potential for having clinical efficacy for protothecosis when given PO or as a retention enema.

## CONFLICT OF INTEREST DECLARATION

Authors declare no conflict of interest.

## OFF‐LABEL ANTIMICROBIAL DECLARATION

Authors declare no off‐label use of antimicrobials.

## INSTITUTIONAL ANIMAL CARE AND USE COMMITTEE (IACUC) OR OTHER APPROVAL DECLARATION

Authors declare no IACUC or other approval was needed.

## HUMAN ETHICS APPROVAL DECLARATION

Authors declare human ethics approval was not needed for this study.

## Supporting information


**Table S1.** Details of the 28 *Prototheca* sp. strains isolated from cases of protothecosis in dogs.

**Table S2.** Minimum inhibitory concentrations (MICs) and minimum algicidal concentrations (MACs) of drugs tested on 28 *Prototheca* sp. isolates.

## References

[1] Jagielski T , Lagneau PE . Protothecosis: a pseudofungal infection. J Mycol Méd. 2007;17:261‐270.

[2] Krüger W . Beitrage zur Kenntnis der Organismen des Saftflusses (sog. Schleimflusses) der Laubbaum. II. Uber zwei aus Saftflussen rein gezuchtete Algen. Zopf's Beitr. Physiol Morph Organ. 1894;4:69‐116.

[3] Jagielski T , Bakuła Z , Gawor J , et al. The genus *Prototheca* (Trebouxiophyceae, Chlorophyta) revisited: implications from molecular taxonomic studies. Algal Res. 2019;43:101639.

[4] Jagielski T , Iskra M , Bakuła Z , et al. Occurrence of *Prototheca* microalgae in aquatic ecosystems with a description of three new species, *Prototheca fontanea*, *Prototheca lentecrescens*, and *Prototheca vistulensis* . Appl Environ Microbiol. 2022;88:e01092‐22. 36300932 10.1128/aem.01092-22 PMC9680628

[5] Jagielski T , Gawor J , Bakuła Z , Decewicz P , Maciszewski K , Karnkowska A . *Cytb* as a new genetic marker for differentiation of *Prototheca* species. J Clin Microbiol. 2018;56:e00584‐18. 30068534 10.1128/JCM.00584-18 PMC6156311

[6] Falcaro C , Furlanello T , Binanti D , et al. Molecular characterization of *Prototheca* in 11 symptomatic dogs. J Vet Diagn Invest. 2021;33:156‐161. 33272142 10.1177/1040638720976423 PMC7758685

[7] Irrgang A , Murugaiyan J , Weise C , Azab W , Roesler U . Well‐known surface and extracellular antigens of pathogenic microorganisms among the immunodominant proteins of the infectious microalgae *Prototheca zopfii* . Front Cell Infect Microbiol. 2015;5:67. 26484314 10.3389/fcimb.2015.00067 PMC4586511

[8] Gmyterco VC , Jagielski T , Baldasso G , Bacher LH , Ribeiro MG , de Farias MR . Cutaneous protothecosis in a dog successfully treated with oral itraconazole in pulse dosing. Acta Vet Scand. 2023;65:7. 36810141 10.1186/s13028-022-00662-x PMC9945405

[9] Silveira CS , Cesar D , Keating MK , et al. A case of *Prototheca zopfii* genotype 1 infection in a dog (*Canis lupus familiaris*). Mycopathologia. 2018;183:853‐858. 29872935 10.1007/s11046-018-0274-5

[10] Stenner VJ , MacKay B , King T , et al. Protothecosis in 17 Australian dogs and a review of the canine literature. Med Mycol. 2007;45:249‐266. 17464846 10.1080/13693780601187158

[11] Van Kruiningen HJ , Garner FM , Schiefer B . Protothecosis in a dog. Protothecosis in a Dog Pat Vet. 1969;6:348‐354. 10.1177/030098586900600404 5388552

[12] Macartney L , Rycroft AN , Hammil J . Cutaneous protothecosis in the dog: first confirmed case in Britain. Ver Rec. 1988;123:494‐496. 10.1136/vr.123.19.494 3201697

[13] Ginel PJ , Pérez J , Molleda JM , Lucena R , Mozos E . Cutaneous protothecosis in a dog. Vet Rec. 1997;140:651‐653. 9226849 10.1136/vr.140.25.651

[14] Ribeiro MG , Rodrigues de Farias M , Roesler U , et al. Phenotypic and genotypic characterization of *Prototheca zopfii* in a dog with enteric signs. Res Vet Sci. 2009;87:479‐481. 19520405 10.1016/j.rvsc.2009.04.015

[15] Sobukawa H , Kano R , Ito T , et al. *In vitro* susceptibility of *Prototheca zopfii* genotypes 1 and 2. Med Mycol. 2011;49(2):222‐224. 20795764 10.3109/13693786.2010.511285

[16] Jagielski T , Buzzini P , Lassa H , et al. Multicentre Etest evaluation of *in vitro* activity of conventional antifungal drugs against European bovine mastitis *Prototheca* spp. isolates. J Antimicrob Chemother. 2012;67:1945‐1947. 22523316 10.1093/jac/dks134

[17] Buzzini P , Turchetti B , Branda E , et al. Large‐scale screening of the *in vitro* susceptibility of *Prototheca zopfii* towards polyene antibiotics. Med Mycol. 2008;46:511‐514. 18608920 10.1080/13693780801993611

[18] Sonne L , Conceição de Oliveira E , Froner Argenta F , et al. *Prototheca zopfii* genotype 2 disseminated infection in a dog with neurological signs. Cienc Rural. 2017;47:e20160877.

[19] Tsuji H , Kano R , Hirai A , et al. An isolate of *Prototheca wickerhamii* from systemic canine protothecosis. Vet Microbiol. 2006;118:305‐311. 16987617 10.1016/j.vetmic.2006.08.003

[20] Dziurzyński M , Decewicz P , Iskra M , Bakuła Z , Jagielski T . Prototheca‐ID: a web‐based application for molecular identification of *Prototheca* species. Database. 2021;2021:baab073. 34791104 10.1093/database/baab073 PMC8607299

[21] CLSI (Clinical and Laboratory Standard Institute) . Reference method for broth dilution antifungal susceptibility testing of yeasts: approved standard. *CLSI* 2008; 3rd ed. Document M27–A3.

[22] Jagielski T , Bakuła Z , di Mauro S , et al. A comparative study of the *in vitro* activity of iodopropynyl butylcarbamate and amphotericin B against *Prototheca* spp. isolates from European dairy herds. J Dairy Sci. 2017;100:7435‐7445. 28711267 10.3168/jds.2017-12597

[23] Jagielski T , Roeske K , Bakuła Z , et al. A survey on the incidence of *Prototheca* mastitis in dairy herds in Lublin province. Poland J Dairy Sci. 2019;102(1):619‐628. 30447976 10.3168/jds.2018-15495

[24] Chen J , Hu X , Li G , et al. Investigation of *Prototheca bovis* infection and its correlation with dairy herd improvement data from a dairy farm in central China. Vet Sci. 2024;11(1):37. 38250943 10.3390/vetsci11010037 PMC10820511

[25] Huilca‐Ibarra MP , Vasco‐Julio D , Ledesma Y , et al. High prevalence of *Prototheca bovis* infection in dairy cattle with chronic mastitis in Ecuador. Vet Sci. 2022;9(12):659. 36548820 10.3390/vetsci9120659 PMC9784310

[26] Endo S , Sekiguchi M , Kishimoto Y , et al. The first case of feline *Prototheca wickerhamii* infection in Japan. J Vet Med Sci. 2010;72(10):1351‐1353. 20460834 10.1292/jvms.09-0504

[27] Kessell AE , McNair D , Munday JS , Savory R , Halliday C , Malik R . Successful treatment of multifocal pedal *Prototheca wickerhamii* infection in a feline immunodeficiency virus‐positive cat with multiple Bowenoid *in situ* carcinomas containing papillomaviral DNA sequences. J Feline Med Surg Open Rep. 2017;3(1):205511691668859. 10.1177/2055116916688590 PMC5415299 28491447

[28] Dillberger JE , Homer B , Daubert D , Altman NH . Protothecosis in two cats. J Am Vet Med Assoc. 1988;192(11):557‐1559. 3410772

[29] Kaplan W , Chandler FW , Holzinger EA , Plue RE , Dickinson RO . Protothecosis in a cat: first recorded case. Med Mycol. 1976;14(3):281‐286. 10.1080/00362177685190421 996693

[30] Finnie JW , Coloe PJ . Cutaneous protothecosis in a cat. Aust Vet J. 1981;57(6):307‐308. 7316900 10.1111/j.1751-0813.1981.tb05832.x

[31] Camboim EKA , Garino FJ , Dantas AFM , et al. Protothecosis by *Prototheca wickerhamii* in goats. Mycoses. 2011;54(4):e196‐e200. 20337944 10.1111/j.1439-0507.2010.01864.x

[32] Macedo JTSA , Riet‐Correa F , Dantas AFM , Simões SVD . Cutaneous and nasal protothecosis in a goat. Vet Pathol. 2008;45(3):352‐354. 18487492 10.1354/vp.45-3-352

[33] Todd JR , King JW , Oberle A , et al. Protothecosis: report of a case with 20‐year follow‐up, and review of previously published cases. Med Mycol. 2012;50(7):673‐689. 22571772 10.3109/13693786.2012.677862

[34] Lass‐Flörl C , Mayr A . Human protothecosis. Clin Microbiol Rev. 2007;20:230‐242. 17428884 10.1128/CMR.00032-06 PMC1865593

[35] Jagielski T , Krukowski H , Bochniarz M , et al. Prevalence of *Prototheca* spp. on dairy farms in Poland—a cross‐country study. Microb Biotechnol. 2019;12(3):556‐566. 30891936 10.1111/1751-7915.13394 PMC6465227

[36] Lassa H , Jagielski T , Malinowski E . Effect of different heat treatments and disinfectants on the survival of *Prototheca zopfii* . Mycopathologia. 2011;171:177‐182. 20853028 10.1007/s11046-010-9365-7

[37] Marques S , Silva E , Carvalheira J , Thompson G . Short communication: temperature sensibility of *Prototheca blaschkeae* strains isolated from bovine mastitic milk. J Dairy Sci. 2010;93:5110‐5113. 20965325 10.3168/jds.2010-3249

[38] Proskurnicka A , Żupnik K , Bakuła Z , Iskra M , Rösler U , Jagielski T . Drug susceptibility profiling of *Prototheca* species isolated from cases of human protothecosis. Antimicrob Agents Chemother. 2023;67(4):e01627‐22. 36943065 10.1128/aac.01627-22 PMC10112244

[39] Jo Siu WJ , Tatsumi Y , Senda H , et al. Comparison of *in vitro* antifungal activities of efinaconazole and currently available antifungal agents against a variety of pathogenic fungi associated with onychomycosis. Antimicrob Agents Chemother. 2013;57:1610‐1616. 23318803 10.1128/AAC.02056-12 PMC3623347

[40] Tortorano AM , Prigitano A , Dho G , Piccinini R , Dapra V , Viviani MA . *In vitro* activity of conventional antifungal drugs and natural essences against the yeast‐like alga *Prototheca* . J Antimicrob Chemother. 2008;61:1312‐1314. 18339634 10.1093/jac/dkn107

[41] United States Food and Drug Administration (FDA) . FDA‐approved drugs. Accessed January 26, 2024. https://www.accessdata.fda.gov/scripts/cder/daf/index.cfm

[42] Watanabe S , Tsubouchi I , Okubo A . Efficacy and safety of fosravuconazole L‐lysine ethanolate, a novel oral triazole antifungal agent, for the treatment of onychomycosis: a multicenter, double‐blind, randomized phase III study. J Dermatol. 2018;45(10):1151‐1159. 30156314 10.1111/1346-8138.14607 PMC6220848

[43] Gupta AK , Leonardi C , Stoltz RR , Pierce PF , Conetta B , the Ravuconazole onychomycosis group . Ravuconazole onychomycosis group. A phase I/II randomized, double‐blind, placebo‐controlled, dose‐ranging study evaluating the efficacy, safety and pharmacokinetics of ravuconazole in the treatment of onychomycosis. J Eur Acad Dermatol Venereol. 2005;19(4):437‐443. 15987289 10.1111/j.1468-3083.2005.01212.x

[44] Hur MS , Park M , Jung WH , Lee YW . Evaluation of drug susceptibility test for efinaconazole compared with conventional antifungal agents. Mycoses. 2019;62(3):291‐297. 30427072 10.1111/myc.12870

[45] Gupta AK , Elewski BE , Sugarman JL , et al. The efficacy and safety of efinaconazole 10% solution for treatment of mild to moderate onychomycosis: a pooled analysis of two phase 3 randomized trials. J Drugs Dermatol. 2014;13(7):815‐820. 25007364

[46] Allgoewer I , Ehrlein J , Goebel T , et al. Disseminated protothecosis in a giant schnauzer. Kleintierpraxis. 1998;43:375‐391.

[47] Cocchetto A , Briola C , Furlanello T , Danesi P , Cirla A , Menchetti M . 3‐T MRI of protothecosis encephalic lesions in a Scottish shepherd dog. Vet Rec Case Rep. 2020;8:e001145.

[48] Enders B , Olby N , Mariani CL . Use of posaconazole for treatment of disseminated protothecosis in a dog. Vet Rec Case Rep. 2016;4:e000350.

[49] Moore FM , Schmidt GM , Desai D , Chandler FW . Unsuccessful treatment of disseminated protothecosis in a dog. J Am Vet Med Assoc. 1985;186:705‐708. 3988604

[50] Rallis T , Tontis D , Adamama‐Moraitou K , Mylonakis M , Papazoglou L . Protothecal colitis in a German shepherd dog. Aus Vet J. 2002;80:406‐408. 10.1111/j.1751-0813.2002.tb10996.x 12222600

[51] Spampinato MF , Kujman S , Cantón J , Daglio C , Catena M . Prototecosis canina: primer reporte en Argentina. Rev Vet. 2017;28:168‐171.

[52] Strunck E , Billups L , Avgeris S . Canine protothecosis. Compend Contin Educ Vet. 2004;26:96‐102.

[53] Alves AC , Morandi S , Cremonesi P , et al. *In vitro* algicidal effect of guanidine on *Prototheca zopfii* genotype 2 strains isolated from clinical and subclinical bovine mastitis. Lett Appl Microbiol. 2017;64:419‐423. 28349671 10.1111/lam.12737

[54] Jagielski T , Niedźwiecka K , Roeske K , Dyląg M . 3‐Bromopyruvate as an alternative option for the treatment of protothecosis. Front Pharmacol. 2018;9:375. 29725298 10.3389/fphar.2018.00375 PMC5917324

[55] Aigner M , Lass‐Flörl C . Encochleated amphotericin B: is the oral availability of amphotericin B finally reached? J Fungi. 2020;6:66. 10.3390/jof6020066 PMC7344640 32443486

[56] Desai JV , Urban A , Swaim DZ , et al. Efficacy of cochleated amphotericin B in mouse and human mucocutaneous candidiasis. Antimicrob Agents Chemother. 2022;66:e00308‐e00322. 35699443 10.1128/aac.00308-22 PMC9295580

