# Supplementary material for: Genotyping and drug susceptibility profiling of Prototheca sp. strains isolated from cases of protothecosis in dogs
Source: J Vet Intern Med. 2024 Nov 20;39(1):e17173. doi: 10.1111/jvim.17173 (PMC11627519; doi:10.1111/jvim.17173)
Supplement: Supplementary file 2 — Table S2. Minimum inhibitory concentrations (MICs) and minimum algicidal concentrations (MACs) of drugs tested on 28 Prototheca sp. isolates. [file JVIM-39-e17173-s001.docx]

**Suppl. Table 2.** Minimum inhibitory concentrations (MICs) and minimum algicidal concentrations (MACs) of drugs tested for 28 *Prototheca* sp. isolates.

| Drug | MIC [mg/L] | | | | | | | |
| --- | --- | --- | --- | --- | --- | --- | --- | --- |
|  | *P bovis* (*n*=21) | | *P ciferrii* (*n*=2) | | *P wickerhamii* (*n*=5) | | *Prototheca* sp. (*n*=28) | |
|  | Range | Median | Range | Median | Range | Median | Range | Median |
| AMB | 0.25 – 8 | 2 | 1 – 8 | 4.5 | 0.5 – 2 | 1 | 0.25 – 8 | 1 |
| EFZ | 0.031 – 0.5 | 0.25 | 0.016 – 0.063 | 0.039 | 0.008 – 0.031 | 0.016 | 0.008 – 0.5 | 0.125 |
| FLU | 16 – 128 | 64 | 16 | 16 | 16 – 64 | 32 | 16 – 128 | 48 |
| ITZ | 2 – 128 | 32 | 2 | 2 | 4 – 32 | 16 | 2 – 128 | 32 |
| KTZ | 2 – 32 | 16 | 1 – 2 | 1.5 | 1 – 32 | 4 | 1 – 32 | 16 |
| RVZ | 0.063 – 2 | 0.5 | 0.125 – 0.25 | 0.188 | 0.016 – 32 | 0.031 | 0.016 – 32 | 0.5 |

**Suppl. Table 2.** Minimum inhibitory concentrations (MICs) and minimum algicidal concentrations (MACs) of drugs tested for 28 *Prototheca* sp. isolates (continued).

| Drug | MAC [mg/L] | | | | | | | |
| --- | --- | --- | --- | --- | --- | --- | --- | --- |
|  | *P bovis* (*n*=21) | | *P ciferrii* (*n*=2) | | *P wickerhamii* (*n*=5) | | *Prototheca* sp. (*n*=28) | |
|  | Range | Median | Range | Median | Range | Median | Range | Median |
| AMB | 0.5 – 8 | 2 | 1 – 8 | 4.5 | 1 – 2 | 1 | 0.5 – 8 | 1.5 |
| EFZ | 0.063 – 1 | 0.25 | 0.016 – 0.063 | 0.039 | 0.016 – 0.063 | 0.016 | 0.016 – 1 | 0.125 |
| FLU | 16 – 128 | 64 | 16 | 16 | 16 – 64 | 64 | 16 – 128 | 64 |
| ITZ | 4 – 128 | 32 | 2 | 2 | 4 – 32 | 32 | 2 – 128 | 32 |
| KTZ | 4 – 64 | 16 | 1 – 2 | 1.5 | 1 – 32 | 4 | 1 – 64 | 16 |
| RVZ | 0.125 – 2 | 1 | 0.25 | 0.25 | 0.031 – 32 | 0.031 | 0.031 – 32 | 0.5 |
